# Plasma circulating tumor DNA assessment reveals *KMT2D* as a potential poor prognostic factor in extranodal NK/T-cell lymphoma

**DOI:** 10.1186/s40364-020-00205-4

**Published:** 2020-07-17

**Authors:** Qiong Li, Wei Zhang, Jiali Li, Jingkang Xiong, Jia Liu, Ting Chen, Qin Wen, Yunjing Zeng, Li Gao, Lei Gao, Cheng Zhang, Peiyan Kong, Xiangui Peng, Yao Liu, Xi Zhang, Jun Rao

**Affiliations:** 1 Medical Center of Hematology, Xinqiao Hospital, Army Medical University, Chongqing, China; 2 State Key Laboratory of Trauma, Burns and Combined Injury, Army Medical University, Chongqing, 400037 China

**Keywords:** ENKTL, Circulating tumor DNA, Mutation allele frequency, Minimal residual disease, Prognosis

## Abstract

**Background:**

The early detection of tumors upon initial diagnosis or during routine surveillance is important for improving survival outcomes. Here, we investigated the feasibility and clinical significance of circulating tumor DNA (ctDNA) detection for Extranodal NK/T-cell lymphoma, nasal type (ENTKL).

**Methods:**

The plasma ctDNA assessment was based on blood specimens collected from 65 newly diagnosed patients with ENKTL in the hematology medical center of Xinqiao Hospital. Longitudinal samples collected under chemotherapy were also included. The gene mutation spectrum of ENKTL was analyzed via next generation sequencing.

**Results:**

We found that the most frequently mutated genes were *KMT2D* (23.1%), *APC* (12.3%), *ATM* (10.8%), *ASXL3* (9.2%), *JAK3* (9.2%), *SETD2* (9.2%), *TP53* (9.2%) and *NOTCH1* (7.7%). The mutation allele frequencies of *ATM* and *JAK3* were significantly correlated with the disease stage, and mutated *KMT2D, ASXL3 and JAK3* were positively correlated with the metabolic tumor burden of the patients. Compared with the tumor tissue, ctDNA profiling showed good concordance (93.75%). Serial ctDNA analysis showed that treatment with chemotherapy could decrease the number and mutation allele frequencies of the genes. Compared with PET/CT, ctDNA has more advantages in tracking residual disease in patients. In addition, patients with mutated *KMT2D* had higher expression compared with those with wild type, and mutated *KMT2D* predicted poor prognosis.

**Conclusion:**

Our results unveil the mutation spectrum of ENKTL patients’ plasma, which can be used to monitor the disease status of the patients exactly, and *KMT2D* is the most frequently mutated gene with prognosis prediction value. The application of ctDNA sequencing can provide precision treatment strategies for patients.

**Trial registration:**

This study is registered with chictr.org (ChiCTR1800014813, registered 7 February, 2018-Retrospectively registered).

## Introduction

Extranodal NK/T-cell lymphoma, nasal type (ENTKL), is an aggressive extranodal lymphoma of NK-cell or T-cell lineage, which is highly aggressive and heterogeneous disease, with predominance in males [1–4]. Current treatment strategies (such as the combination of chemotherapy, radiotherapy, targeted therapy) can improve the complete remission of patients, but most will ultimately relapse and progress [5–7]. For these patients, standard monitoring methods, including computed tomography (CT) and serum protein markers analysis upon initial diagnosis and during routine surveillance, are pivotal for therapeutic response evaluation; however, these tests have limitations of low sensitivity and specificity or false positivity. Therefore, there is an urgent need for a highly sensitive, standardized and noninvasive assay that can be used for detecting early relapse and/or progression of disease.

Current treatment response criteria for non-Hodgkin lymphoma (non-HL) rely on CT scans or positron emission tomography (PET) scans. Imaging scans upon initial diagnosis or recurrence can provide macro overviews of the tumor volume and location, but CT scans have some limitations due to cost and radiation exposure, and PET lacks specificity [8–10]. Moreover, imaging scans cannot monitor dynamic tumor response and the clonal evolution in time. Therefore, clinically validated technology is needed to overcome the current monitoring treatment response limitations. One potential biomarker for detection is circulating cell-free DNA (cfDNA), which comes from dying cells that release DNA fragments into circulation, and in tumor patients, some of the cfDNA primarily originates from apoptosis and necrotic cancer cells, which carry tumor-specific alterations (termed circulating tumor DNA, ctDNA) [11–14]. ctDNA sequencing is a promising tool for the real-time monitoring of tumor progression, and its application has been explored in multiple solid tumors [15]. ctDNA can reflect the temporal evolution of tumors. Current studies have shown that ctDNA detection can be used to monitor minimal residual disease and track recurrence after treatment [16, 17]. In addition, ctDNA sequencing is a noninvasive and tumor-specific assay, and ctDNA in circulation has a half-life of less than 2 h, which is more sensitive than protein biomarkers and more specific than routine surveillance imaging through CT scans or PET scans [18]. Thus, dynamic tracking of ctDNA would be of higher value in guiding personalized management strategy.

The clinical application of ctDNA has been performed for multiple types of lymphoma, such as diffuse large B-cell lymphoma (DLBCL), classical HL, follicular lymphoma, multiple myeloma and peripheral T-cell lymphoma [19–25]. ctDNA assessment acts as a dynamic marker of burden during treatment based on immunoglobulin next-generation sequencing (Ig-NGS), T-cell receptor gene next-generation sequencing (TCR-NGS) and cancer personalized profiling sequencing (CAPP-Seq). Recent evidence concerning ctDNA assessment in peripheral T-cell lymphoma by TCR-NGS suggested that ctDNA detection in T-lineage lymphoma is feasible [26]. In the present study, we thoroughly determined the molecular spectrum of ctDNA mutations in ENTKL and characterized the correlation between ctDNA mutations and clinical factors. Paired tumor tissues and plasma samples were collected to compare the concordance between tumor DNA and ctDNA. Moreover, dynamic ctDNA mutation alterations during treatment were also observed.

## Materials and methods

### Study design and patient selection

In this prospective cohort study, 65 patients newly diagnosed with ENKTL in the hematology medical center of Xinqiao Hospital from February 2017 to December 2019 were enrolled (ClinicalTrials identifier: ChiCTR1800014813). All consecutive patients who were deemed appropriate for this study during the study period were included without selection. Histological diagnoses were established independently by at least two experienced senior pathologists according to the WHO classification of Tumors of Hematopoietic and Lymphoid tissue criteria. All patients underwent baseline staging using laboratory, radiographic, and bone marrow examinations upon diagnosis. The Eastern Cooperative Oncology Group (ECOG) performance status was also assessed. The stage was evaluated in accordance with the Ann Arbor staging system. The International Prognostic Index (IPI) was calculated based on the serum lactate dehydrogenase, stage, extranodal status and performance status. Patient characteristics and treatment regimens of each therapy cycle were collected from each patient. Three healthy individuals were also recruited as control participants to test the accuracy of our sequencing platform for ctDNA profiling. All participants provided informed written consent before undergoing any study-related procedures in accordance with the Declaration of Helsinki. This study was approved by the China Ethics Committee of Registering Clinical Trials (ChiECRCT-20,180,005).

After the initial stage assessment, all patients were given 6 to 8 cycles of pegaspargase-based CHOP like regimens. Patients were reviewed routinely by a combination of clinical assessment and CT or fluorodeoxyglucose-PET (FDG-PET) before the administration of chemotherapy. FDG-PET was often used as an interim scan, and the metabolic tumor volume (MTV) was determined from the initial and interim PET images using PET Edge software (MIMSoftware Inc., Cleveland, OH, USA). Serial ctDNA profiling was conducted during the patient’s therapy course.

### Sample collection and DNA extraction

Before treatment, peripheral blood samples were collected using 10 ml EDTA vacutainer tubes and processed within 4 h at the constant temperature of 4 °C. cfDNA was extracted from plasma using the QIAamp Circulating Nucleic Acid Kit (Qiagen, Valencia, California) following the manufacturer’s instructions. Genomic DNA was extracted from peripheral mononuclear cells and formalin fixed paraffin-embedded (FFPE) tissue. The DNA concentration and quality were estimated using a Qubit fluorometer (Invitrogen). The cfDNA quality was assessed using an Agilent 2100 Bioanalyzer and DNA HS Kit (Agilent Technologies, Palo Alto, CA, USA).

### Multiregional targeted NGS of patients’ plasma and tumor sample

Sequencing was performed on FFPE tumor DNA, plasma cfDNA and PBMC, and targeted sequencing gene panels including the coding exons and splice sites of 41 genes (Yuanqi Biopharmaceutical Co., Shanghai, China) that are recurrently mutated in NK/T-cell lymphoma were specifically designed for this study, with the genes including *ADAM3A*, *APC, ARID1A, ARID1B, ARID2, ASXL3, ATM, BCOR, BCORL1, CD28, CHD8, CREBBP, DDX3X, DNMT3A, EP300, EZH2, FYN, IDH2, IL2RG, JAK1, JAK3, KDM6A, KMT2A, KMT2D, MGA, NF1, NOTCH1, PRDM1, PTPN1, RHOA, SETD2, SOCS1, STAT3, STAT5B, STAT6, TET1, TET2, TNFRSF14, TP53, TRAF3* and *ZAP608*. Tumor DNA was sheared through sonication before library construction to obtain an almost 200-bp fragment of cfDNA, which possesses a fragmented DNA nature. No additional fragmentation was performed before library construction. NGS libraries were constructed using the SureSelect Library Prep Kit (Agilent Technologies, Palo Alto, CA, USA). Quantification of the library was performed using the Agilent DNA 1000 Kit (Agilent Technologies). Sequencing was performed on the Illumina MiSeq system (Illumina, San Diego, CA) following the manufacturer’s protocol. For tumor DNA, the mean depth of each sample was 2500×. Lengths of the cfDNA fragments primarily ranged from 100 to 200 bp, and the average coverage depths for sequencing was 813.42× (range, 462 × − 6513×), with an average of 5% of the target sequence being covered at sufficient depth for variant calling. Bioinformatics analysis was performed to verify the sample sequence and mutation site and to calculate the mutated allele frequency (MAF) compared with the human genome sequence (hg19) using Burrows-Wheel Aligner (BWA) sequence alignment software. Samtools version 1.3 was used for single nucleotide variant (SNV)/indel calling and filter workflow.

### Statistical analysis

Statistical analysis was conducted using SPSS software (Version 18.0, LEAD Corp). Descriptive statistics were used to analyze the clinical, demographic and genetic test result characteristics. Concentrations of ctDNA were expressed in haploid genome equivalents per mL (hGE/mL) and were calculated by multiplying the mean ctDNA mutant allele frequencies by the input concentration of cfDNA in pg/mL as determined by fluorometry and then dividing by 3.3 [20]. The correlation of gene mutation, ctDNA concentration and clinicopathologic features of patients were conducted using the Pearson χ^2^ tests. The overall survival (OS) measured the proportion of patients who were alive at a specific time after diagnosis. Survival estimates were obtained using the Kaplan–Meier method, and comparisons were made using a log-rank test. Unpaired Student’s t-test for two groups was used in this study. A COX proportional regression model was used to calculate the survival hazard ratio (HR). Statistical differences were considered significant if the *P* value was less than 0.05.

## Results

### Patient characteristics

**Fig. 1:**
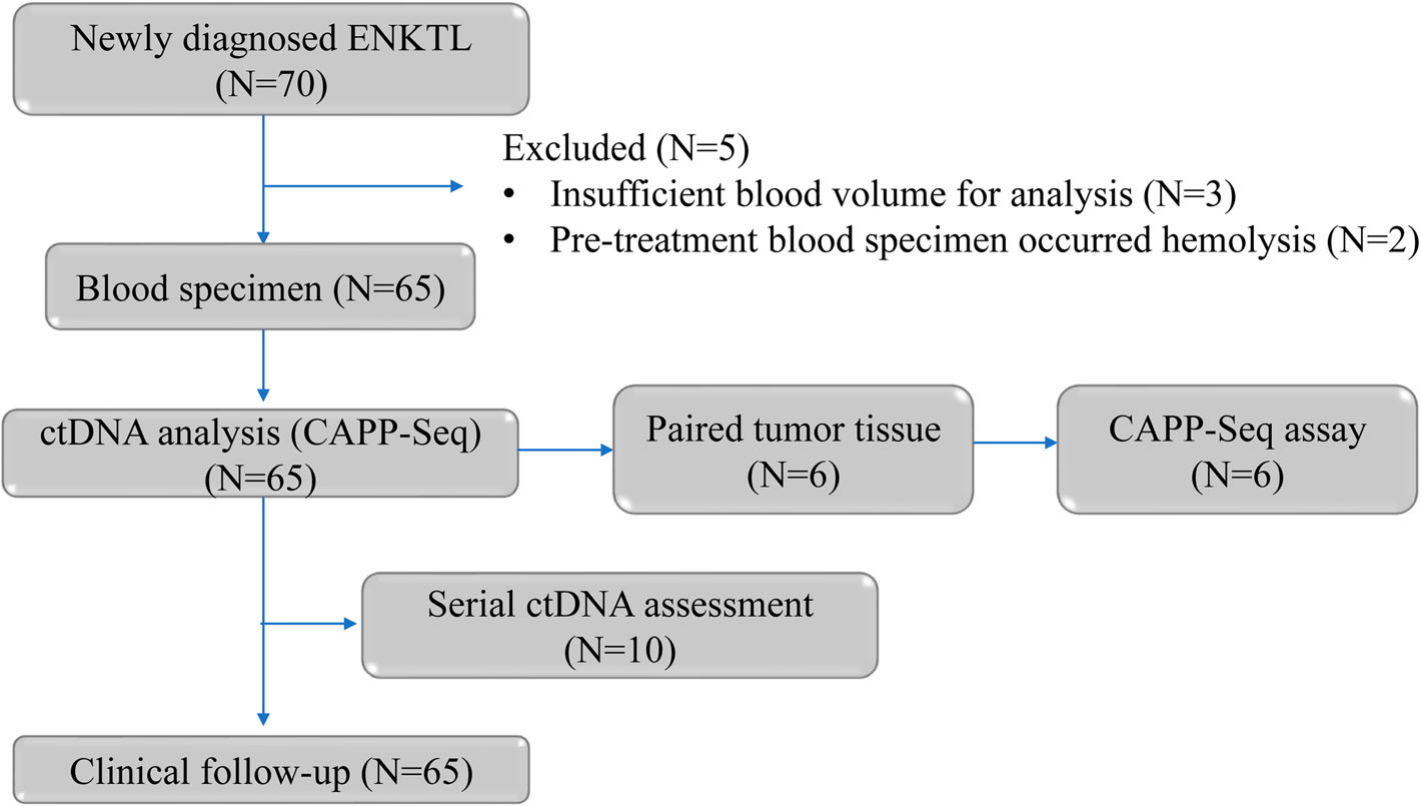
Consort diagram of patient enrolment and specimen collection

To examine the feasibility of ctDNA detection in plasma, from February 2017 to December 2019, 65 newly diagnosed ENKTL patients were recruited, and initial and longitudinal plasma specimens were obtained from patients undergoing chemotherapy (Fig. 1). The median age at first blood draw was 45.2 years (range, 40 to 55 years), and 45 patients were men. At disease onset, 26 patients (40%) exhibited B symptoms. According to the Ann Arbor staging criteria, 14 patients were identified as stage I, 20 patients were identified as stage II, 13 patients were identified as stage III, and 18 patients were identified as stage IV (Table 1). ctDNA was successfully extracted from all patients and healthy individuals. The average concentration of the ctDNA was 3.79 hEG/ml (range, 0 to 5.39 hEG/ml).

**Table 1:** Patients’ demographic and clinical characteristics

Prognostic variables	No.	Proportion(%)
**Gender**		
Male	45	69.23
Female	20	30.77
**Clinical stage**		
I	14	21.54
II	20	30.77
III	13	20.00
IV	18	27.69
**B symptoms**		
With	26	40.00
Without	39	60.00
**IPI Scores**		
1	22	33.85
2	24	36.92
3	14	21.54
4	5	7.69
**Final Recurrence status**		
With	9	13.85
Without	56	86.15
**Ki67 index**		
Low	33	50.77
High	32	49.23

### ctDNA mutation spectrum of newly diagnosed ENKTL

**Fig. 2:**
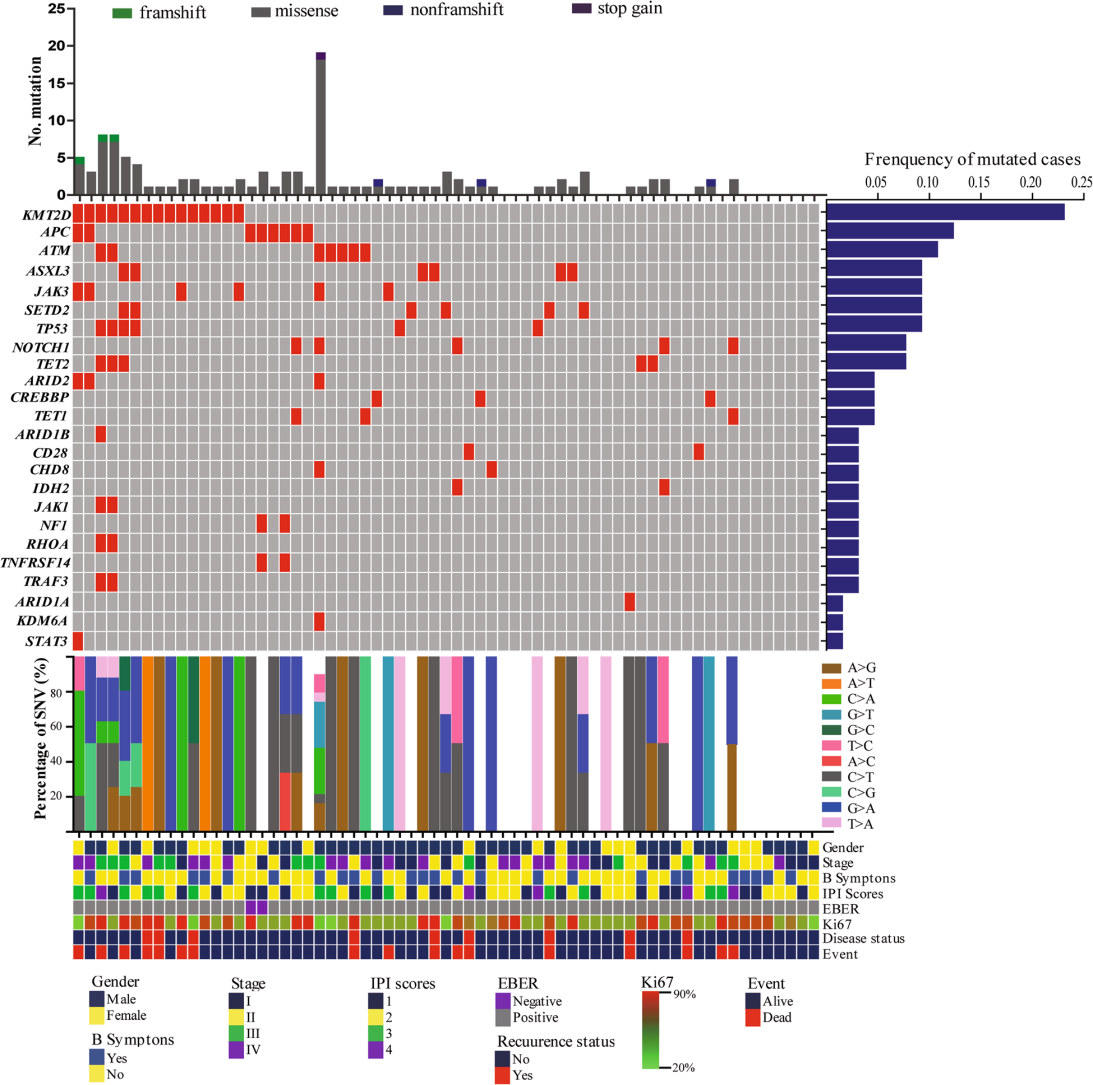
The mutational profile of newly diagnosed ENKTL. The heatmap shows individual nonsynonymous somatic mutations detected in the ctDNA of newly diagnosed patients (*n* = 65). Each row represents a gene, and each column represents a primary tumor. Mutations are color coded in red. The upper bar graph shows the number and type of mutated genes, the horizontal bar graph shows the gene mutation frequency, the middle bar graph shows the percentage of nonsynonymous somatic mutations, and the lower graph shows the clinical characteristics of each sample

Of the 65 patients recruited in this study, to exclude hematopoietic clonal mutations, somatic mutations observed in both plasma and PBMC were excluded, mutation were detected in the plasma of 49 (75.3%) patients with a median of 1.7 per sample (range, 0 to 19) (Fig. 2, upper panels). The range of mutant allele frequencies in each gene of the samples was shown in Table S1. The most frequently mutated genes were *KMT2D* (23.1%), *APC* (12.3%), *ATM* (10.8%), *ASXL3* (9.2%), *JAK3* (9.2%), *SETD2* (9.2%), *TP53* (9.2%) and *NOTCH1* (7.7%) (Fig. 2, middle panels). Consistent with the somatic SNV spectrum in other tumors, we found that C > T/G > A was a preferred alteration (Fig. 2, lower panels).

### Correlation of detectable ctDNA with the clinical characteristics of ENKTL patients

**Fig. 3:**
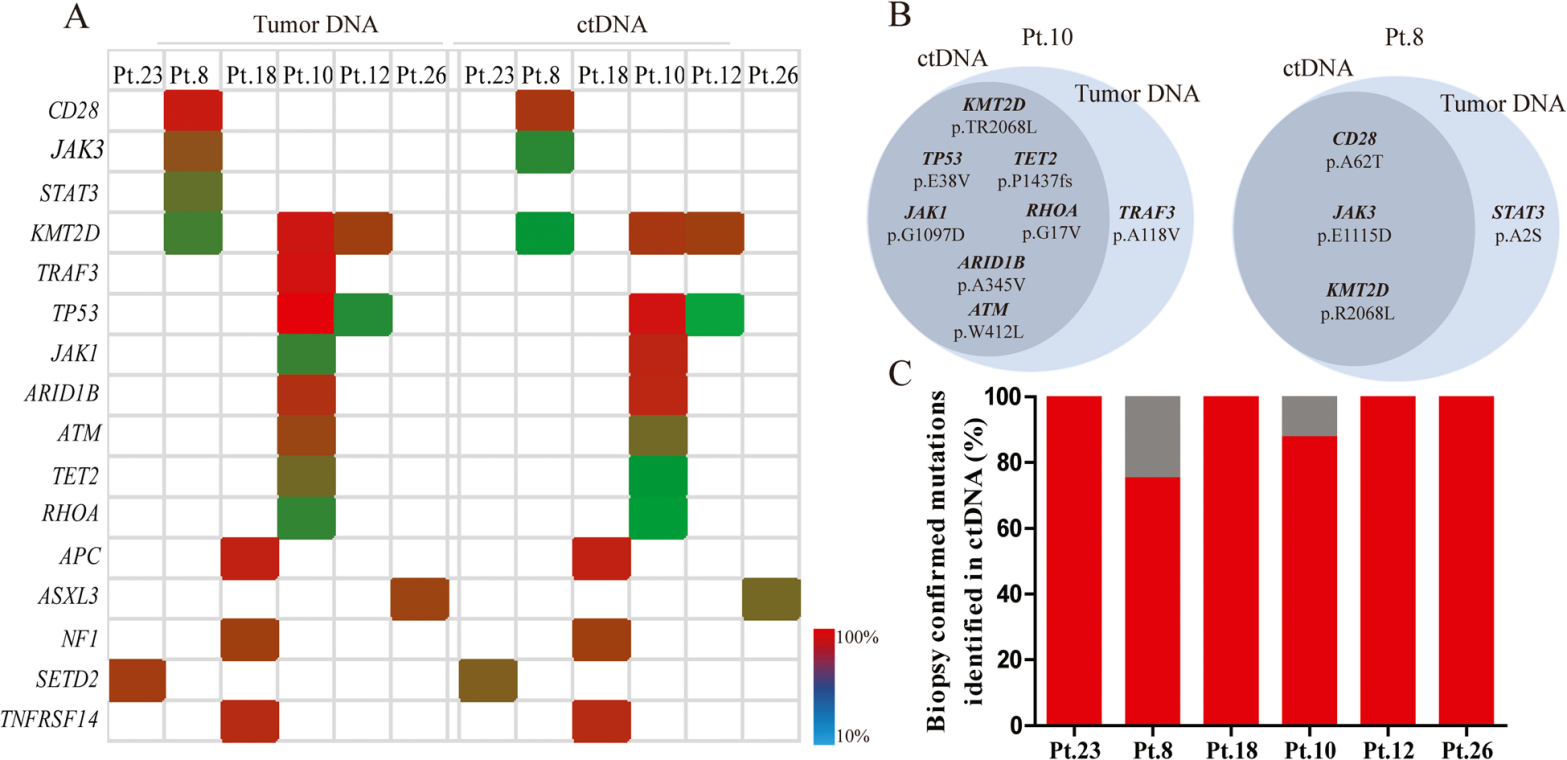
Concordance of the ctDNA assessment with the tissue NGS test. **a** The consistent gene mutation spectrum and mutation allele frequency detected in the plasma ctDNA and tumor tissue of the patients. **b** Venn diagram summarizing the detailed mutations discovered in both plasma ctDNA (gray) and tumor DNA (blue). **c** For each patient, the fraction of tumor biopsy-confirmed mutations that were detected in plasma ctDNA is shown. The gray portion of the bar marks the part of the tumor biopsy-confirmed mutation that was not found in the plasma ctDNA

To assess the concordance of the ctDNA test with the tissue NGS test, 6 paired tumor biopsies were genotyped. Compared with the results of the plasma ctDNA spectrum, most of the genes overlapped, the average biopsy-confirmed detection ratio was 93.75% (range 75–100%) (Fig. 3a). In 4 patients, biopsy-confirmed tumor mutations were all detectable in ctDNA samples (Fig. 3b), but for patient #10, *TRAF3* variation was not detected in tumor DNA. For patient #8, *STAT3* could not be detected in tumor ctDNA (Fig. 3c). These differences might be caused by tumor anatomical heterogeneity or tumor-associated stromal tissue infiltration.

**Fig. 4:**
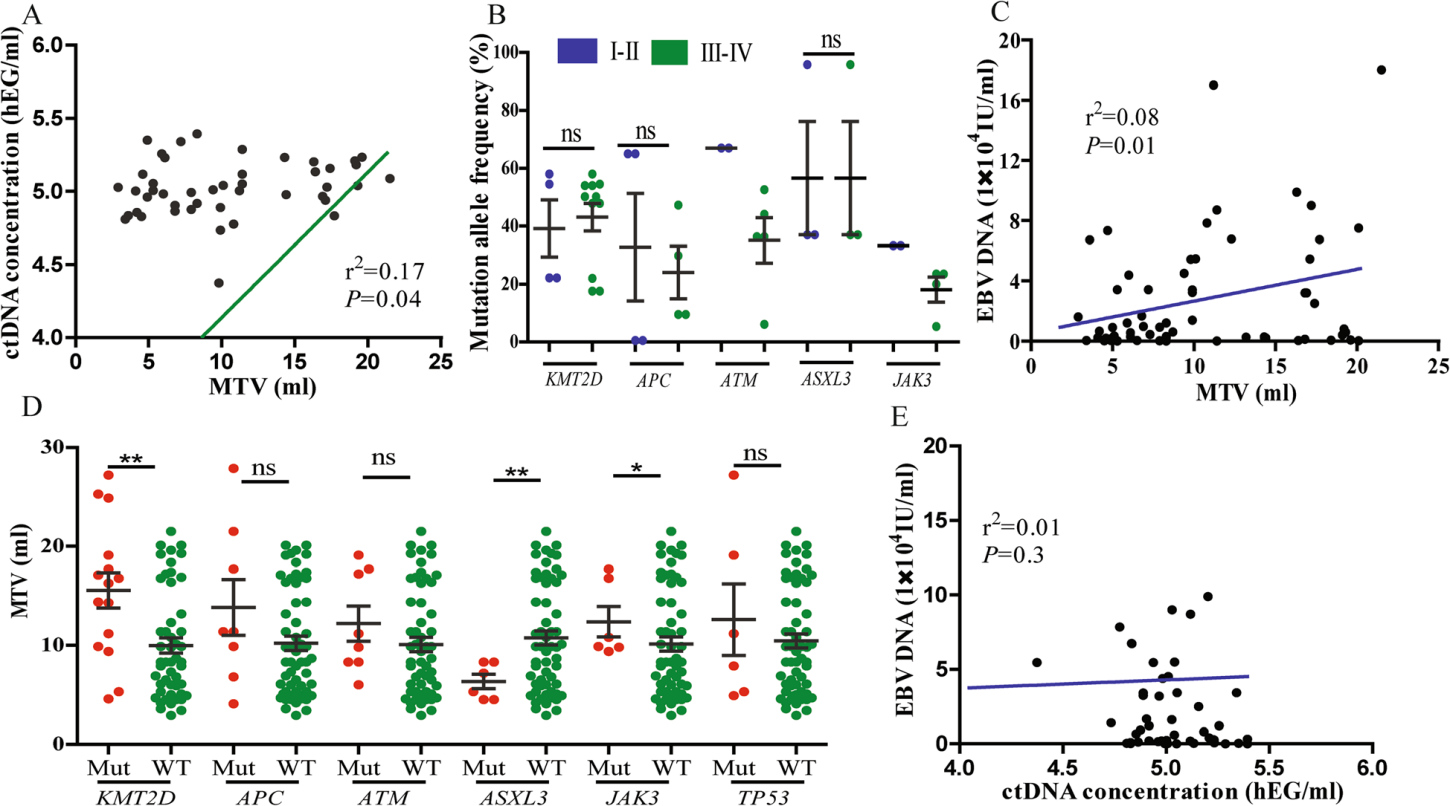
Correlation of the ctDNA assessment with the patient clinical characteristics. **a** Linear regression of the plasma ctDNA concentration with metabolic tumor volume. **b** Mutation allele frequencies of *KMT2D, APC, ATM, ASXL3 and JAK3* in patients with different tumor stages. **c** The correlation between plasma EBV-DNA levels and metabolic tumor volume. **d** The general metabolic tumor volume in patients with different mutation statuses of *KMT2D, APC, ATM, ASXL3, JAK3* and *TP53*. **e** The correlation of the EBV-DNA level with the ctDNA concentration

To validate the potential clinical utility of the mutations detected in the plasma ctDNA, we investigated the correlation between clinical factors and ctDNA levels. As MTV measured by PET/CT could quantitatively reflect the tumor burden, we found that the plasma ctDNA concentration was significantly correlated with the MTV (*P* = 0.04) (Fig. 4a), suggesting that ctDNA could be identified as a promising biomarker of the tumor load. In patients with stage III-IV disease, the mutation frequencies of *KMT2D* (11/33), *ATM* (5/33), and *JAK3* (3/33) were higher than that of patients with stage I-II disease. Additionally, mutation allele frequencies of *KMT2D, APC* and *ASXL3* were not correlated with disease stage, but the mutation allele frequencies of *ATM* and *JAK3* showed significant differences (Fig. 4b). Furthermore, the correlation of the mutation status of each gene with the metabolic tumor burden was investigated. We found that patients with mutated *KMT2D, ASXL3,* and *JAK3* showed significantly higher MTV than patients with wild type (Fig. 4d), suggesting that these genes might be positively correlated with the disease malignancy. Recently, plasma EBV-DNA has served as a valuable biomarker of the tumor load and prognostic factor, thus, we also evaluated the correlation between the plasma EBV-DNA and clinical characteristics, and found that the pre-treatment plasma EBV-DNA was positively correlated with MTV (Fig. 4c), but the correlation between EBV-DNA levels and the ctDNA concentration were not significant different (Fig. 4e).

### Serial ctDNA detection during therapy could complement the response assessment of the patients, and patients with mutated *KMT2D* and *ATM* predicted poor prognosis

**Fig. 5:**
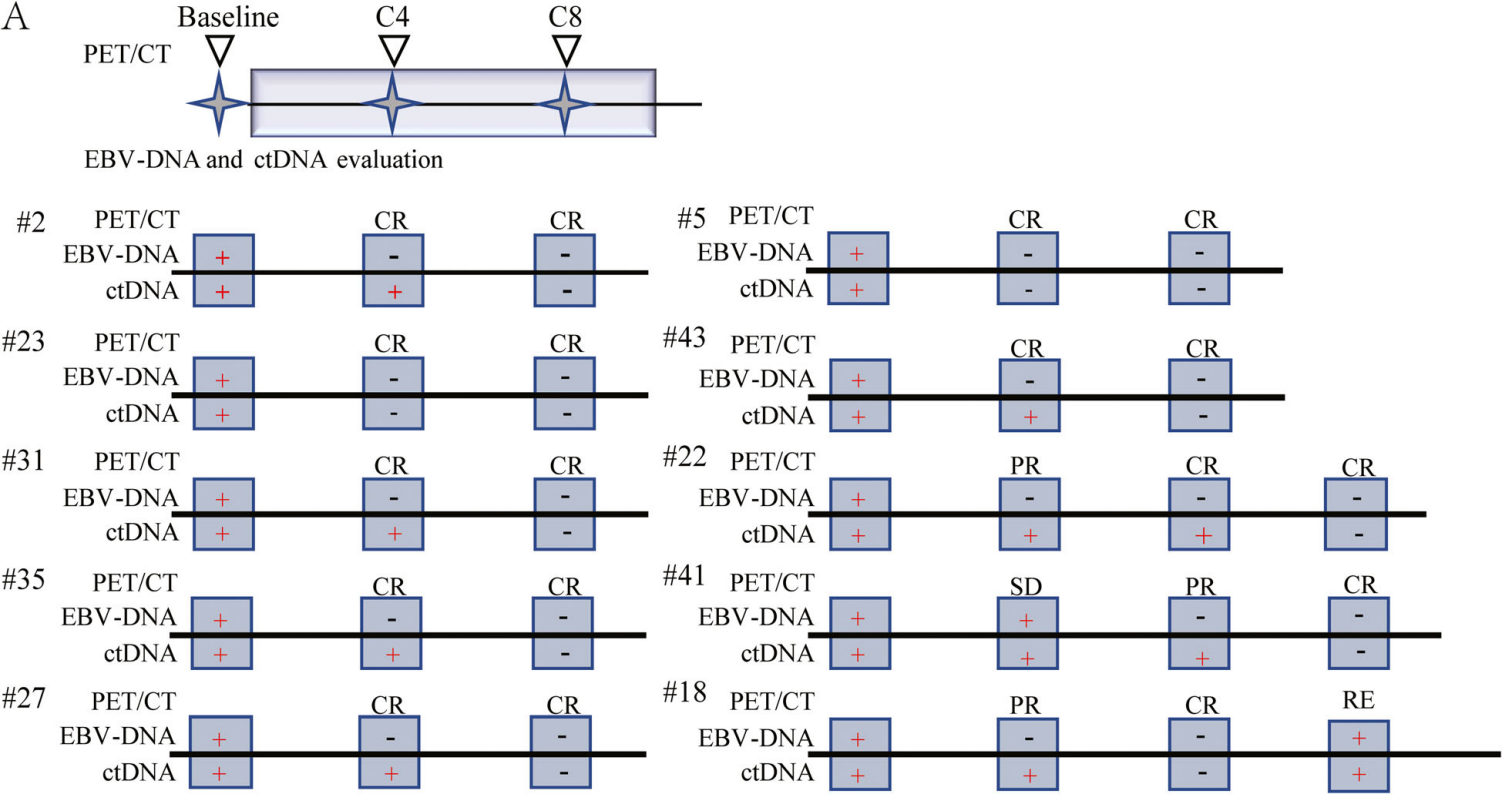
Serial ctDNA detection during therapy could complement the response assessment of the patients. Treatment response assessment using PET/CT was combined with genomic testing of the ctDNA and plasma EBV-DNA levels. Plasma and imaging were performed at baseline prior to treatment initiation, before the start of cycle 4 (C4), and before cycle 8 (C8). Displayed are recruited patients with serial plasma collection and their responses to treatment using the plasma EBV-DNA, ctDNA assessment and PET/CT scans

We further investigated the potential role of plasma ctDNA in the therapeutic monitoring of patients undergoing chemotherapy. Ten patients were recruited, and sequential plasma samples upon disease diagnosis, before treatment cycle 4 (C4), and before cycle 8 (C8) were collected. In total, the mutated gene number and mutation allele frequencies decreased with the chemotherapy administration, 7 patients could achieve CR at C4, in these patients, only 2 patients didn’t detect ctDNA mutations at C4, suggesting that ctDNA assessment was more sensitive than PET/CT scans, all patients couldn’t detect plasma EBV-DNA when achieved CR. In addition, with more cycles of chemotherapy, all patients could achieve complete molecular remission (Fig. 5). In these 10 patients, we also observed one relapsed, and the relapsed mutation spectrum was similar to the original spectrum. All these results suggested that ctDNA detection could be regarded as a molecular response evaluation marker, and could complement the response assessment of the patients.

**Fig. 6:**
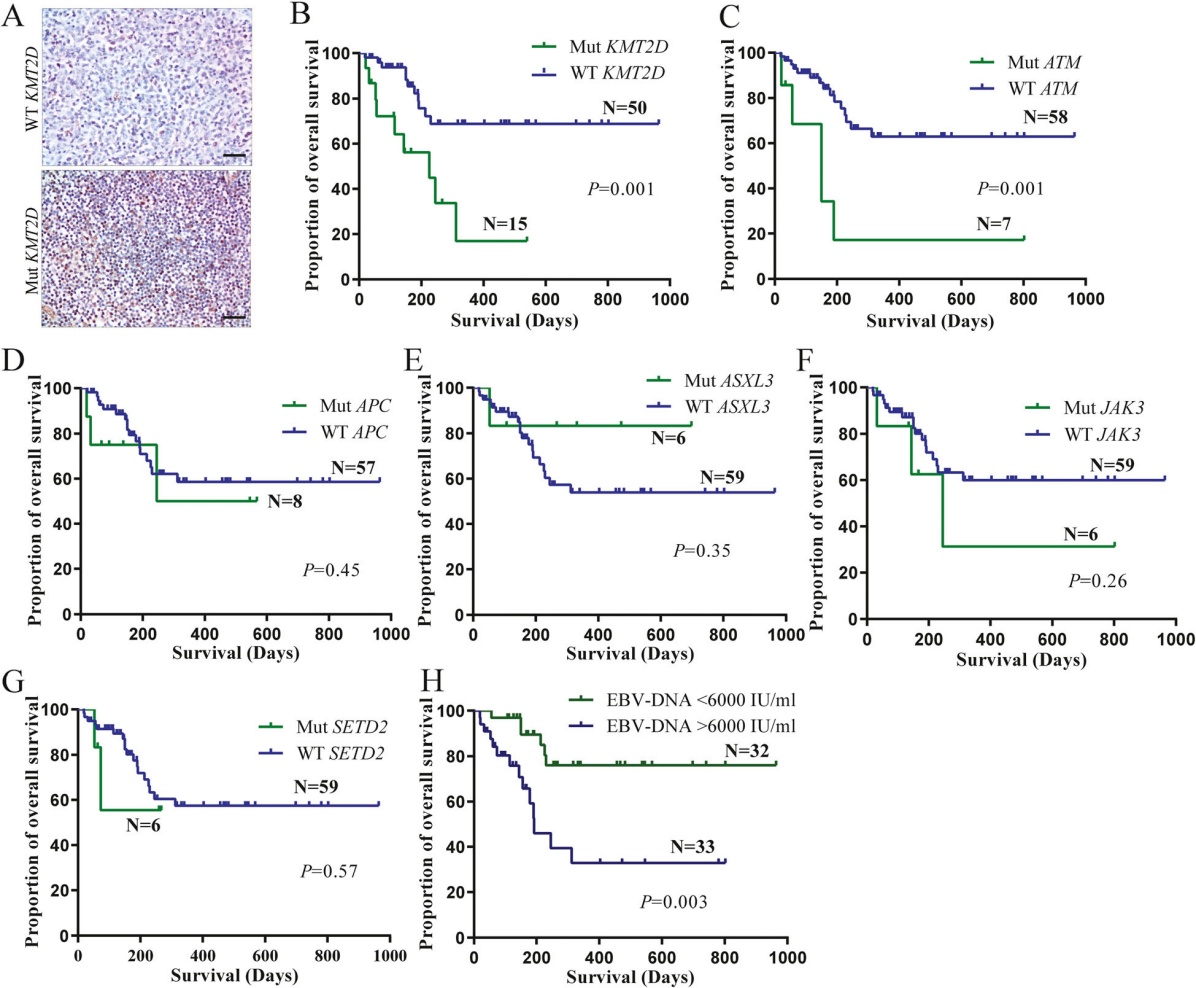
Prognostic value of frequently mutated genes for patients with ENKTCL. **a** Immunohistopathology staining of KMT2D in patients with mutated KMT2D and wild type, scale bar, 25 μm. Kaplan-Meier estimated OS with the plasma *KMT2D* (**b**), *ATM* (**c**), *APC* (**d**), *ASXL3* (**e**), *JAK3* (**f**), *SETD2* (**g**) mutation status in patients with ENKTL, and plasma EBV-DNA levels (**h**)

As *KMT2D* was the most frequently somatic mutations, the expression of KMT2D was investigated, and we found that the KMT2D expressions of patients with mutations were higher than that of patients with wild type (Fig. 6a). Then, prognosis value of the plasma ctDNA mutation status and EBV-DNA level was also investigated. Kaplan-Meier analysis estimated that patients with mutated *KMT2D* and *ATM* had a shorter OS (Fig. 6b, c), but no significant difference was observed in patients with other mutated genes (Fig. 6d-g), which might be due to the small sample recruited in our cohort. In addition, patients with high EBV-DNA levels showed significantly poorer prognosis (Fig. 6h). The results of univariate and multivariate analysis for risk factor of OS in patients were summarized in Table 2. Univariate analysis of the factors revealed that the stage, IPI scores, recurrence status, EBV-DNA levels, *KMT2D* mutation and *ATM* mutation were independent prognostic indicators of the overall survival of patients, and multivariate analysis showed that the EBV-DNA level, *KMT2D* mutation and *ATM* mutation were solely prognostic factors. All these results suggested that the dynamic detection of ctDNA during therapy could better reflect the disease status than PET/CT scan, and patients with mutated *KMT2D,* and *ATM* predicted poor prognosis.

**Table 2:** Univariate and multivariate analyses of the overall survival of patients

Prognostic variables	Univariate analysis		Multivariate analysis	
	HR (95% CI)	*p value*	HR (95% CI)	*p value*
Gender (female vs. male)	0.712 (0.279–1.812)	0.476		
Stage (I-II vs. III-IV)	2.625 (1.043–6.604)	0.04	1.159 (0.253–5.311)	0.849
B symphony (with vs. without)	1.234 (0.512–2.973)	0.64		
IPI	2.595 (1.073–6.277)	0.034	0.565 (0.118–2.712)	0.476
Final recurrence status	3.346 (1.364–8.204)	0.008	2.705 (0.941–7.776)	0.065
MTV	2.069 (0.843–5.078)	0.112		
ctDNA concentration	2.975 (0.984–8.997)	0.053		
EBV-DNA level	3.672 (1.396–9.664)	0.008	3.174 (1.091–9.231)	0.034
*KMT2D* mutation (without vs. with)	0.289 (0.119–0.702)	0.006	0.266 (0.086–0.828)	0.022
*APC* mutation (without vs. with)	0.550 (0.16–1.895)	0.344		
*ATM* mutation (without vs. with)	0.271 (0.098–0.752)	0.012	0.212 (0.064–0.700)	0.011
*ASXL3* mutation (without vs. with)	2613 (0.347–19.68)	0.351		
*JAK3* mutation (without vs. with)	0.569 (0.166–1.949)	0.369		
*SETD2* mutation (without vs. with)	0.804 (0.184–3.507)	0.771		
*TP53* mutation (without vs. with)	0.615 (0.179–2.115)	0.441		
*NOTCH1* mutation (without vs. with)	0.410 (0.120–1.406)	0.156		

## Discussion

This study was the first to determine the plasma ctDNA gene mutation spectrum of ENKTL and demonstrated that ctDNA sequencing could be considered a promising biomarker for monitoring the minimal residual disease (MRD) of patients. The most frequently mutated genes in plasma ctDNA were *KMT2D* (23.1%), *APC* (12.3%), *ATM* (10.8%), *ASXL3* (9.2%), *JAK3* (9.2%), *SETD2* (9.2%), *TP53* (9.2%), and *NOTCH1* (7.7%). The ctDNA concentration was positively correlated with tumor stage and MTV. In terms of consistency with tumor tissue, almost all of the gene mutations detected in the plasma ctDNA could be found in tumor DNA, and further analysis demonstrated that a decreased number and MAF of certain genes in plasma ctDNA could complement the therapeutic response of the patients, and patients with mutated *KMT2D* and *ATM* had poor prognosis, suggesting that the gene mutations in plasma could serve as promising biomarkers for use in the diagnosis or monitoring of ENKTL disease courses.

Tumor biopsies can only offer information about lymphoma at one specific time in a specific area and can be accompanied with complications, and PET/CT may lead to false negatives due to technology limitations or false positive caused by tumor flares or pseudoprogression [27]. ctDNA detection is an optimal noninvasive and specific technique by which to overcome these traditional monitoring method limitations. A rapidly growing body of evidence has established that ctDNA detection during curative therapies can identify patients for whom there remains evidence of residual, radiographically occult cancer. In DLBCL, ctDNA can be used to predict the tumor load and treatment outcome of newly diagnosed patients, and patients with a pretreatment ctDNA level above the median had a significantly poorer prognosis than those with levels below the median [28]. Later, Kurtz et al. reported that serial ctDNA measurements of 125 patients were an effective tool for prognostication, patients with 2-log or greater decreases in ctDNA had a better event-free survival (EFS) after one cycle of therapy, and patients with 2.5-log or greater reduction in ctDNA had a significantly better EFS after two cycle treatments. These studies suggested that serial ctDNA detection could be used to assess the therapy response dynamically and guide personalized target therapy. In another study, Daigle et al. found that mutation of *CREBBP* in R/R DLBCL had the greatest association with tazemetostat treatment (an EZH2 inhibitor), and patients with mutations in *PIM1*, *BCL6*, *HIST1H1E* and *TP53* lacked response to treatment [29]. In HL, Valeria et al. determined the molecular mutation spectrum using ctDNA measurement. The most frequently mutated genes were *STAT*, *TNFAIP3* and *ITPKB*, and detection sensitivity was 87.5%, suggesting that ctDNA could well reflect the spectrum of tumor tissue mutation. Compared with newly diagnosed and refractory HL mutation profiles, most mutations overlapped, and mutations of *STAT*, *TNFAIP3, GNA13* and *ITPKB* might be an ancestral clone persisted throughout the disease course [23]. Although only a few studies have focused on assessing ctDNA in T-cell lymphoma, preliminary evidence has also demonstrated that ctDNA measurement in TCL is feasible [26, 30]. Our study is the first to determine the mutation spectrum of ctDNA in ENKTL. The most commonly mutated genes are *KMT2D, APC, ATM, ASXL3, JAK3, SETD2, TP53* and *NOTCH1*. Our results revealed that ctDNA could reflect the MTV, and by longitudinally profiling patients treated with chemotherapy. In addition, we provided evidence that serial ctDNA assessment could monitor the disease status, with the results able to partially reflect which mutated gene was sensitive to chemotherapy drugs. However, only one of the 10 recruited patients relapsed. For relapsed patients, whether primary mutation clone recurrence or new mutation occupies the main role needs to be further investigated. In addition, future trials should include more patients and long-term follow-up to explore the plasma gene clone evolution process.

Plasma cfDNA includes tumor cell-derived plasma ctDNA and normal cell apoptosis-derived cfDNA, which are DNA fragments. ctDNA could be a more comprehensive reflection of gene mutations in all tumors than conventional biopsies [31, 32]. Therefore, ctDNA-based detection methods are critical for identifying which mutation are tumor-specific. As sensitivities increase with the development of new methods and because age-related somatic mutations could also lead to hematopoietic clonal expansion, the possibility of false-positive results might occur [33, 34]. Recently, Razavi et al. showed that clonal hematopoiesis was the major contributor to plasma variants using a high-intensity sequencing assay of matched plasma DNA, tumor tissue and white blood cells, emphasizing that matched cfDNA-white blood cells were critical for accurately screening somatic mutations [35]. In the present study, to decrease the bias caused by clonal hematopoiesis associated mutations, the age range of the included patients was narrow, matched ctDNA-PBMC sample was applied, and a sequencing panel was conducted according to a previous gene-expression profiling study on ENKTL. Zhao et al. confirmed that the most frequently mutated genes in NKTCL were the RNA helicase gene *DDX3X*, tumor suppressors (*TP53* and *MGA*), JAK-STAT pathway molecules (*STAT3* and *STAT5b*) and epigenetic modifiers (*MLL2, ARID1A, EP300* and *ASXL3*) [36, 37]. In addition to these recurrent mutations, *JAK3, KMT2D, EZH2, NOTCH1* and *TET2* were also identified by different groups [38–41]. Based on these findings combined with the most frequent gene mutation spectrum in Chinese individuals, we selected 41 mutated genes for our study. Interestingly, we found that *KMT2D* alterations possessed a higher frequency (23.1%) and which were all non-synonymous mutations (p.R2068L, p.D4378E, p.R755W, p.P2382S, p.V160L, p.L2316V). We also found some mutation existed in both plasma and PBMC, such as *MGA* (p.T716S, p.P1523A), *ASXL3* (p.N954S), *EZH2* (p.D185H), *TET2* (p. P29R), *P53* (p.P72R), *APC* (p.V1822D), *KDM6A* (p.T726K), et al. KMT2D belongs to a family of mammalian histone H3 lysine 4 (H3K4) methyltransferase. It is frequently mutated in Kabuki syndrome and various cancers, and it acts as a scaffold protein within the complex depending on the cell type and stage of differentiation [42]. In acute myeloid leukemia, follicular lymphoma and DLBCL, KMT2D could inhibit tumorigenesis and metastasis [43, 44], but in solid cancer, such as prostate cancer, KMT2D is critical factor for promoting tumor cell proliferation [45]. In our study, we found that the KMT2D expression of patients with the mutation was higher than that of patients with wild type, suggesting that KMT2D might be an oncogene in NKTCL lymphoma. However, its underlying significance in NKTCL has not been explored to date, and further studies regarding the function of *KMT2D* mutation in NKTCL lymphomagenesis are urgently recommended.

## Conclusions

To our knowledge, this is the first study to prospectively evaluate the potential utility of ctDNA analysis for NKTCL patients. We have demonstrated that ctDNA assessment could predict the therapy response in NKTCL, and the highest mutation frequencies were in *KMT2D*, *APC*, *ATM*, *ASXL3*, *JAK3*, *SETD2*, *TP53*, *NOTCH1*. Our results could define the optimal strategy for patient follow-up.

## Supplementary information

**Additional file 1: Table S1.** The mutant allele frequencies in each gene of each sample.

**Additional file 2: Table S2.** Correlation between *KMT2D* mutation status and clinicopathological features of ENKTL.

**Additional file 3: Table S3.** Correlation between *ATM* mutation status and clinicopathological features of ENKTL.

## Data Availability

All data generated and analyzed during this study are included in this article and its supplementary information files.

## Abbreviations

ENTKL: Extranodal NK/T-cell lymphoma, nasal type
CT: Computed tomography
Non-NHL: Non-Hodgkin lymphoma
cfDNA: Circulating cell-free DNA
ctDNA: Circulating tumor DNA
DLBCL: Diffuse large B-cell lymphoma
MTV: Metabolic tumor volume
ADAM3A: ADAM Metallopeptidase Domain 3A
*APC*: Adenomatous Polyposis Coli Protein
*ARID1A*: AT-Rich Interaction Domain 1A
*ARID1B*: AT-Rich Interaction Domain 1B
*ARID2*: AT-Rich Interaction Domain 2
*ASXL3*: ASXL Transcriptional Regulator 3
*ATM*: Ataxia Telangiectasia Mutated
*BCOR*: BCL6 Corepressor
*BCORL1*: BCL6 Corepressor Like 1
*CHD8*: Chromodomain Helicase DNA Binding Protein 8
*CREBBP*: CREB Binding Protein
*DDX3X*: DEAD-Box Helicase 3 X-Linked
*DNMT3A*: DNA Methyltransferase 3 Alpha
*EP300*: E1A Binding Protein P300
*EZH2*: Enhancer of Zeste 2 Polycomb Repressive Complex 2 Subunit
*FYN*: Src Family Tyrosine Kinase
*IDH2*: Isocitrate Dehydrogenase (NADP (+)) 2, Mitochondrial
*IL2RG*: Interleukin 2 Receptor Subunit Gamma
*JAK1*: Janus Kinase 1
*JAK3*: Janus Kinase3
*KDM6A*: Lysine Demethylase 6A
*KMT2A*: Lysine Methyltransferase 2A
*KMT2D*: Lysine Methyltransferase 2D
*MGA*: MAX Dimerization Protein
*NF1*: Neurofibromin 1
*NOTCH1*: Notch Receptor 1
*PRDM1*: Positive Regulatory Domain I-Binding Factor 1
*PTPN1*: Protein Tyrosine Phosphatase Non-Receptor Type 1
*RHOA*: Ras Homolog Family Member A
*SETD2*: SET Domain Containing 2
*SOCS1*: Suppressor of Cytokine Signaling 1
*STAT3*: Signal Transducer and Activator of Transcription 3
*STAT5B*: Signal Transducer and Activator of Transcription 5B
*STAT6*: Signal Transducer and Activator of Transcription 6
*TET1*: Tet Methylcytosine Dioxygenase 1
*TNFRSF14*: TNF Receptor Superfamily Member 14
*TP53*: Tumor Protein P53
*TRAF3*: TNF Receptor Associated Factor 3
*ZAP608*: Zeta Chain Of T Cell Receptor Associated Protein Kinase 608
MAF: Mutated allele frequency
SNV: Single nucleotide variant
