# Supplementary material for: Plasma circulating tumor DNA assessment reveals KMT2D as a potential poor prognostic factor in extranodal NK/T-cell lymphoma
Source: Biomark Res. 2020 Jul 17;8:27. doi: 10.1186/s40364-020-00205-4 (PMC7366898; doi:10.1186/s40364-020-00205-4)
Supplement: Supplementary file 2 — Additional file 2: Table S2. Correlation between KMT2D mutation status and clinicopathological features of ENKTL. [file 40364_2020_205_MOESM2_ESM.docx]

| Supplementary Table 2 Correlation between *KMT2D* mutation status and clinical characteristics | | | | | |
| --- | --- | --- | --- | --- | --- |
| Prognostic variables | No. | *KMT2D*(WT) | *KMT2D*(Mut) | χ2 | *P* |
| **Gender** |  |  |  |  |  |
| Male | 45 | 36 | 9 | 0.78 | 0.38 |
| Female | 20 | 14 | 6 |  |  |
| **clinical stage** |  |  |  |  |  |
| I-II | 34 | 29 | 5 | 2.814 | 0.09 |
| III-IV | 31 | 21 | 10 |  |  |
| **B symptoms** |  |  |  |  |  |
| with | 20 | 14 | 6 | 0.78 | 0.38 |
| without | 39 | 36 | 9 |  |  |
| **IPI Scores** |  |  |  |  |  |
| 1 | 22 | 19 | 3 | 6.449 | 0.09 |
| 2 | 24 | 18 | 6 |  |  |
| 3 | 14 | 9 | 5 |  |  |
| 4 | 5 | 4 | 1 |  |  |
| **Recurrence status** |  |  |  |  |  |
| with | 9 | 6 | 3 | 0.619 | 0.43 |
| without | 56 | 44 | 12 |  |  |
| **Ki67 index** |  |  |  |  |  |
| Low | 33 | 27 | 6 | 0.905 | 0.34 |
| High | 32 | 23 | 9 |  |  |
| **EBV-DNA copy** |  |  |  |  |  |
| Low | 31 | 24 | 7 | 0.008 | 0.93 |
| High | 34 | 26 | 8 |  |  |
